# “You need to take care of it like you take care of your soul”: perceptions and behaviours related to mosquito net damage, care, and repair in Senegal

**DOI:** 10.1186/1475-2875-13-322

**Published:** 2014-08-15

**Authors:** Dana K Loll, Sara Berthe, Sylvain L Faye, Issa Wone, Bethany Arnold, Hannah Koenker, Joan Schubert, Youssoufa Lo, Julie Thwing, Ousmane Faye, Rachel Weber

**Affiliations:** Center for Communication Programs, Johns Hopkins Bloomberg School of Public Health, Baltimore, MD USA; Department of Sociology, University Cheikh Anta DIOP, Dakar, Senegal; Department of Public Health, University Cheikh Anta DIOP, Dakar, Senegal; Center for Communication Programs, Johns Hopkins Bloomberg School of Public Health, Dakar 4, Senegal; Centers for Disease Control and Prevention, Atlanta, GA USA; Department of Animal Biology, University Cheikh Anta Diop, Dakar, Senegal

**Keywords:** Malaria, Insecticide-treated net, Long-lasting insecticidal net, Durability, Integrity, Net care, Net repair

## Abstract

**Background:**

Net care and repair behaviours are essential for prolonging the durability of long-lasting insecticidal nets. Increased net durability has implications for protection against malaria as well as cost savings from less frequent net distributions. This study investigated behaviours and motivations for net care and repair behaviours in Senegal with the aim of informing social and behaviour change communication (SBCC) programmes, using the Health Belief Model as a framework.

**Methods:**

Data were collected from 114 participants in eight regions of Senegal. Participants were eligible for the study if they were at least 18 years old and if their household owned at least one net. These respondents included 56 in-depth interview respondents and eight focus groups with 58 participants. In addition, the qualitative data were supplemented with observational questionnaire data from a total of 556 sleeping spaces. Of these spaces, 394 had an associated net.

**Results:**

Reported net care and repair behaviours and motivations varied substantially within this sample. Children and improper handling were seen as major sources of net damage and respondents often tried to prevent damage by storing nets when not in use. Washing was seen as an additional method of care, but practices for washing varied and may have been damaging to nets in some cases. Participants mentioned a sense of pride of having a net in good condition and the uncertainty around when they could expect another net distribution as motivations for net care. Net repair appeared to be a less common behaviour and was limited by the perspective that net degradation was inevitable and that repairs themselves could weaken nets.

**Conclusion:**

These findings can be understood using the Health Belief Model framework of perceived severity, perceived susceptibility, perceived barriers, perceived benefits, self-efficacy, and cues to action. This model can guide SBCC messages surrounding net care and repair to promote practices associated with net longevity. Such messages should promote the benefits of intact nets and provide tools for overcoming barriers to care and repair.

## Background

Malaria remains a leading cause of morbidity and mortality in endemic countries. Long-lasting insecticidal nets (LLINs) have been proven effective in reducing malaria transmission by preventing contact between the vector and the human host as well as control of the vector itself [1]. Therefore, the distribution and promotion of LLINs has been integrated into malaria control programmes throughout the world [2]. While net distributions previously targeted specific vulnerable populations (infants and pregnant women), efforts now focus on achieving universal coverage, defined at the household level as the use of insecticide-treated nets by all household members, regardless of age or gender [3]. Through mass distribution campaigns, access to nets has increased but still falls short of universal coverage goals [2]. As soon as nets are distributed, they begin a process of inevitable degradation, developing holes and tears and losing insecticide over time.

In the context of continually shifting resources for distribution of LLINs [4] and inevitable wear and tear of nets, it is important to understand the factors at the household level that affect net durability as well as perceptions and behaviours towards net damage, care and repair. By clearly defining perceptions and experiences with net care and repair, it may be possible to develop better methods and approaches to encourage behaviours consistent with preventing damage to nets and repairing any damage that occurs. As a result, nets may be more likely to remain intact for a longer amount of time before becoming ineffective and needing replacement.

### Net durability

The durability and useful life of LLINs varies widely and is context and care dependent [5–15]. WHO guidelines for monitoring the durability of LLINs under operational conditions state that net durability should be measured both in terms of physical or fabric integrity and insecticidal activity or bio-efficacy [16].

Factors, including the ecological conditions, the frequency of washing, the age of net, the sleeping surface, the way the net is used, the number of people who sleep under it, and the denier of the fabric can affect the condition of a net and how long it lasts [8]. The number of washes and location of drying are correlated with the amount of residual insecticide; nets that are washed less frequently and dried in the shade retain more insecticide than those that are washed frequently and dried in the sun [17, 18]. Other documented causes of net damage include tears on bed frames and mattresses, fire, domestic animals, rats, toenails, drying, children playing, and contact between the net and the walls of the house [12, 13].

Nets with many holes are often perceived to be useful no longer [6, 19, 20]. In Ethiopia, the major reason given for non-use of existing nets was that they were too torn [19]. In Kenya, Mutuku and colleagues showed that bed net use decreases with increasing physical damage of the net. In a previous study, it was shown that user-determined end of net life was defined largely by the condition of the net and the presence of mosquitoes in the net due to holes [20]. Observational studies show that more holes in the net make the net less effective at preventing mosquito entry [12, 21].

### Net care

In order to avoid and address net degradation, it is important to understand care and repair practices. Washing is typically seen as the main form of caring for one’s net. The World Health Organization Pesticide Evaluation Scheme (WHOPES) minimum standards indicate that procured nets must be able to maintain insecticidal activity for at least 20 washes [16]. Yet existing evidence shows that in some cases nets are washed quite frequently, as often as every two to three weeks [22]. Even when net users are provided with information about the recommended washing frequency, it can be challenging for them to adhere to this advice due to dust, children soiling the nets, or the idea that sleeping under a net that is not clean is bad for one’s health [22–24]. Miller and colleagues suggested that social pressures for cleanliness and concerns about the health implications of sleeping under a dirty net made less frequent washing unacceptable to the respondents [22]. Mutuku and colleagues showed that the frequency of washing was correlated with the age and colour of the net, with newer nets being washed less frequently [13]. Since frequent net washing is associated with degraded physical condition and diminished insecticide content [11, 13], understanding the barriers to a reduction in washing frequency is critically important.

### Net repair

Once nets are damaged, it is important for owners to repair the nets both in terms of longevity of net life and protection from malaria. The existing body of evidence, however, indicates that net repair is a relatively uncommon practice and even when done, nets are most often not completely repaired [6, 9, 13]. One study in Ethiopia found that only about 4% of households were making repairs to their nets [6]. While net repair was shown to be more common in Ghana and Kenya, nets were rarely completely repaired and repair did not appear to improve the overall condition of the nets [9, 13]. In Ghana, repairs were most commonly done using needle and thread [9]. In Kenya, the female head of household most commonly repaired nets and repairs were most often conducted in households that owned only one net [13].

Net care and repair practices are, in principle, likely to increase net durability and integrity. The condition of a net appears to be correlated with the likelihood that it will be used [6, 19, 20] as well as its effectiveness for prevention of malaria [12, 21]. In a context of competing health priorities and limited funding, it is essential that people use their nets for as long as possible and take measures to prolong their lifespan.

The Health Belief Model (HBM) [25], a theory of behaviour change that has proved useful in interpreting malaria behaviours in previous studies [26, 27], is used here as a framework. HBM constructs include perceived susceptibility, perceived severity, perceived benefits, perceived barriers, self-efficacy, and cues to action (such as presence of mosquitoes), which combine and lead to specific behaviour action (e.g., net care and/or repair). Self-efficacy and cues to action were added more recently to help explain maintenance of behaviours [28]. This article identifies causes of net damage at the household level and describes attitudes and perceptions around net care and repair behaviours in Senegal. It will also consider how these factors might be integrated into social and behaviour change communication (SBCC) programmes to encourage net care and repair practices that are consistent with improved useful net life.

## Methods

### Study sites

Data were collected in eight regions of Senegal in order to allow for analysis of variations due to geographical, cultural, and LLIN coverage differences. In each region, a rural and a peri-urban site were selected for data collection.

### Study population and procedures

The data were collected during the second phase of a two-phase study. Findings from the first phase informed discussion topics for the second phase. The analysis and results presented in this paper focus on the second phase of research.

Data were collected during the height of the rainy season by four data collection teams. Each team was responsible for two regions. All researchers were trained on the study objectives, study design and ethical treatment of human subjects and were accompanied by a supervisor in the field during data collection.

Participants in the in-depth interviews (IDIs) and focus group discussions (FGDs) were eligible for the study if their compound owned at least one net and if they were over the age of 18. Once these eligibility criteria were verified, the head of compound and the IDI participant were asked to provide consent to participate. Study team members then drew a map of the compound, noting all structures and habitual sleeping spaces, as well as those used during the previous night. The map also noted whether there was a net associated with each individual sleeping space. Data collectors were trained to probe about alternative places where family members may sleep beyond those that were visible. Each sleeping space was assigned a number, which correlated to a questionnaire about that sleeping space. The sleeping space questionnaires collected information on presence of a net for that sleeping space, the condition of the net, who slept in the space during the previous night, and behaviours related to caring for and repairing the net. The sleeping space questionnaire respondents were anyone in the household who had knowledge of the sleeping space or the person (if over 18) who used it during the previous night. Someone who was a typical user of the sleeping space most often completed the questionnaire. IDIs were conducted with a randomly selected adult member of the household in order to maximize the perspectives attained in the study. Other eligible adults in the selected household were eligible to be recruited for the FGD. FGD participants were recruited from participating and newly entering households in each community with the help of community leaders. IDI and FGD interview guides covered a range of topics related to barriers and motivators for net use, perceptions and practices of net care and repair, and how people decide that a net is no longer useful.

A total of eight focus groups were conducted in four of the eight regions and participants were purposively sampled from the selected communities based on age, sex, and net ownership. FGDs were homogenous by sex and ranged from six to nine participants. There were a total of 11 refusals to participate in the study, mostly among peri-urban participants who did not have the time needed for the interview. There were seven refusals among the IDI participants and four refusals among FGD participants; these refusals were replaced with other participants. The total sample size was 114 participants including 56 IDI respondents and 58 FGD participants from the eight FGDs. This total sample included perspectives from 54 women and 58 men. In two IDIs, the sex of the respondent was not recorded. Of the 56 households in the study, 24 of them had previously been visited in the first phase of the study. Sleeping space questionnaires were collected on a total of 556 sleeping spaces, 394 of which had nets associated with them.

### Data analysis

Interviews and discussions were conducted and audiorecorded in local languages including Wolof, Pulaar and Serere, and then transcribed and translated verbatim into French in Microsoft Word. These textual data were entered into ATLAS.ti and coded by a team of four independent coders using a codebook of themes of interest. Coding was conducted using a primarily deductive approach while allowing for the addition of emergent codes and themes. The coders met frequently throughout the coding, to ensure that codes were being applied in the same way and to discuss the addition of codes as themes arose from the data. The analysis focused on overall perspectives and regional variations regarding the ways that nets become damaged and methods and motivations for caring for and repairing nets.

### Ethical considerations

Ethical approval for each phase of research was secured from the Johns Hopkins University Bloomberg School of Public Health Institutional Review Board in Baltimore, USA and from the *Comité National d’Ethique pour la Recherche en Santé* in Dakar, Senegal. Participants provided oral consent prior to participating in the study.

## Results

### Perceived causes of net damage

The reported causes of net damage included daily or improper use of nets, the act of sleeping restlessly or with too many people under a net, the act of hanging a net, negligent behaviour with the net, the actions of children, washing the net, the environment surrounding the net, pests, and wear and tear associated with net repair. Results regarding the most noteworthy causes of damage are presented below.

### Daily/improper net use

The reported primary source of net damage, across all regions, was daily and/or improper net use. ‘Improper use’ included pulling the net too tight while hanging it, having too many people sleeping under a net, not properly untucking it and storing it during the day, and other actions such as being too rough with the net, that could result in net damage. Pulling nets too tightly or roughly, or tearing them on the bed in the process, was mentioned as an especially problematic cause of damage.

R6: *Maybe if it is not well tucked in? And the fact of not arranging it/putting it away when waking up, leaving it always hung up, or perhaps when you move too much while sleeping. These may tear the net.*

R5*: The people who kick around a lot while sleeping. The net, if you respect it, it won’t tear.*

-FGD participants, Kedougou urban, male

Sometimes damage was caused by not caring for or repairing a net, while some respondents labelled damage as being caused by “negligence”. Many respondents generally considered that damage to nets may be reflective of the way a person lives his or her life. These participants noted that there is a social judgment that accompanies a torn net.

R: *If you are negligent, the net may be torn because if you have children who cannot stay calm, they can pull the net and destroy it. That’s why you should not be negligent. Everything related to health should not be neglected.*

-FGD respondent, Thies urban, female

### Role of children

Children were frequently blamed for causing damage to nets, either while playing in and around nets and sleeping spaces during the day, or sleeping restlessly at night. Children, as a cause of net damage, were a theme that emerged in every region. Participants reported that children do not understand that nets are fragile and that they can be easily damaged during playing.

R*: Yes, sometimes the children play with (the nets)*

R*: In general, it’s the main cause, meaning they play with the net, they take an object because they are thoughtless*

R*: They may destroy the net without realizing it. If they do not care…that may wear out the nets and make them unusable.*

-IDI respondent, Saint Louis urban

### Over/under washing

Washing a net too frequently was seen as a way to damage a net; at the same time, not washing a net often enough was also seen as potentially damaging. Respondents noted that once nets were washed, they could also be torn on fences and door jams when hung to dry.

### Structural causes of net damage

In Kedougou and Kolda, respondents cited their beds or mattresses as being major causes of net damage, especially if they used or slept on bamboo beds. These participants suggested that the nets frequently get caught on the rough ends of the bamboo and that the construction of the bamboo bed contributed to tears and snags.

R*: Well, frequently it’s the beds, honestly. There are bamboo beds like that. It’s the beds made from bamboo raffés.*

E*: Raffé, ok.*

R*: Yes, the bamboo raffé beds. So if we have beds like that and you put down a net, it’s from there that the tears will come. When you put it under the mattress, during the night you will move, it pulls, how do I say this, the attachment point that also may be the origin of the tears.*

-IDI respondent, Kedougou urban

Some respondents stated that where a net is stored, both seasonally or during the day, could result in damage to nets. Participants suggested that the damage during storage could occur due to mice and other pests or from snagging the net on a nail or other sharp object.

In Dakar and Saint Louis, respondents mentioned sleeping outdoors as potentially damaging to nets, due to the frequent moving of a net to an outdoor sleeping space or being caught in the rain while sleeping outdoors and needing to quickly get inside.

### Weather and seasonal/environmental factors

Weather-related factors were also perceived to contribute to net degradation. This included the role of the sun in damaging nets, both generally and in terms of being laid to dry in the sun after washing. Some mentioned that due to seasonal changes, especially in urban areas, mosquitoes can be found year-round, rather than just a few months a year in the rural areas. This causes people to need to use their nets year-round, and this consistent use of nets contributed to their degradation.

More specifically, the sun was often seen as contributing to damage, but this was only discussed in Saint Louis and Louga and not throughout the country. Some acknowledged that drying the net in the sun after washing may cause damage.

R*: Yes, it also happens that once you attach it, if you are lazy instead of taking off the net, you leave it in the sun, until the sun beats down on it, that may also fatigue the nets. The sun fatigues mosquito nets. Ok, in fact, the sun, if someone is exposed, as a person, you see the effects…The effects that happen to a person are the same for objects.*

-IDI respondent, St. Louis urban

### Other causes of damage

A minority of respondents discussed damage caused from pests, including mice, rats and insects. Finally, a few respondents discussed repairing nets as a way of further damaging them, or perceived that net repair was a never-ending pursuit.

R*: When you sew with thread and a needle, the thread will create a hole as well so there’s no reason to sew it. The needle creates holes, that’s why I took down my net because there was not sense. It only provokes other holes to sew with a needle. Myself, to repair (nets) I put another piece of fabric over my net (old net) but the problem is that it’s hot…and the heat is not good.*

-IDI respondent, Thies urban

### Net care

Participants were asked about how they take care of their nets. In addition to washing and repairing nets, respondents mentioned properly hanging and storing nets, retreating nets with insecticide, avoiding sharp objects, and the seasonal non-use of nets.

### Net care motivations

Many respondents said it is important to take care of a net because it is the net that protects them from sickness and from mosquito nuisance. Respondents cited health benefits, cost savings from averting illness, and sleeping well at night as benefits that resulted from a well cared for or intact net. Respondents discussed protection from malaria as the primary reason for caring for one’s net.

*To take care of a net is to take care of yourself because it protects you from malaria and other illnesses. It protects you in that mosquitoes, flies and germs cannot touch you. For me, to take care of a mosquito net is to protect oneself.*

-IDI respondent, Fatick rural

A few respondents noted that while they had recently received a net in a distribution, it was unclear when they would receive nets again and therefore they felt that they had to care for the ones that they had.

### Net storage

Hanging and storing nets in the “proper manner” was the most commonly mentioned way to care for a net. Respondents in all regions said that nets should be tied up when not in use, or even removed and stored in a bag or a cupboard. Since children were mentioned as a major source of net damage, storing the nets to keep them from playing in or pulling on the nets was considered very important. Respondents said that nets should be put down only during sleeping and then tied up or folded immediately.

R*: If you sleep there until morning, you hang it up. Avoid bringing it down during the day.*

-IDI respondent, Dakar rural

### Net washing

Many respondents discussed washing their nets but ideas of how to do this correctly varied greatly. Most respondents mentioned that nets should be washed “when they become dirty”. The dirt was often said to be a result of dust and was even thought by some to be unsafe for their families. Some respondents said that having a dirty net could transmit disease itself. Others mentioned that dirt could make the nets fragile. These respondents, therefore, mentioned that nets should be washed very frequently.

*If the mosquito net stays for a long time without being washed, the dirt is one thing that tears the nets. If you don’t wash nets enough, they tear very quickly. If the mosquito net is clean, it doesn’t tear very quickly, it can remain useful for years.*

-IDI respondent, Kolda rural

Other respondents, however, expressed concern for the well being of the net due to repeated washings. These respondents reported that nets should not be frequently washed in order to preserve the insecticide and/or the physical integrity of the net.

*Because when you have a mosquito net and you hang it for a month…two months…three, four months, the dust settles above and you have to take it to wash and dry it…that causes lots of holes in the mosquito net.*

*-* IDI respondent, Kedougou urban

Most respondents mentioned net washing frequencies in between these two extremes. Many respondents mentioned that they washed their nets with Omo, a detergent, and fewer mentioned the use of soap and bleach. Since WHOPES criteria are based on the use of regular soap, it is unknown whether the use of Omo, or other harsh detergents may further reduce bio-efficacy of insecticide.

**Figure 1:**
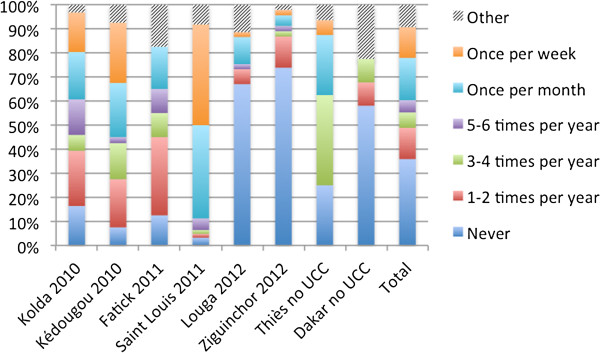
**Frequency of net washing by region.** Date indicates when the region received the universal coverage campaign (UCC). Thies and Dakar received the UCC after the fieldwork for this study was completed.

In the sleeping space questionnaire, respondents were asked to report on how frequently they washed the net associated with a given sleeping space. One third of all nets (35.9%) had reportedly never been washed. The majority of nets in Ziguinchor (73.9%), Louga (67.0%) and Dakar (58.1%) were reported to have never been washed. Ziguinchor and Louga had benefited from the universal coverage campaign most recently, in 2012.Yet many respondents also reported that they washed their nets weekly, monthly or five to six times per year. Figure 1 presents washing frequency by region, as gathered in the IDIs/FGDs/sleeping space. Due to differences in climate and geography, different regions in Senegal are likely to vary in washing frequency due to increased prevalence of dirt and sand. Nets appeared to be most frequently washed in Saint Louis, with 41.9% of respondents reporting that they washed their nets weekly and 38.7% of respondents indicating that they did so monthly. Nets were washed frequently in Kedougou and Kolda, where nets were distributed in 2010.

After washing, many respondents said that they usually dried their nets in the sun. Some even mentioned that by drying nets in the sun, they could eliminate any insects that were living in the nets. However, other respondents reported that nets should be dried in the shade and that this was important for their effectiveness.

R*: After having washed and re-impregnated the net, you do not dry it in the sun. Look for a place where there is shade and put a mat and install the net. It will dry and the product will stay and it will protect you from malaria and all of the mosquitoes that come will die there. And when she (the net distributor) told me, I tested this myself.*

-IDI respondent, Fatick urban

**Figure 2:**
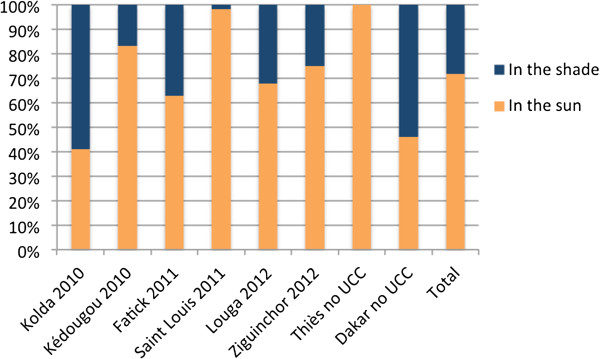
**Reported net drying practices by region.** The net is the unit of analysis. Dates indicate when each region received the universal coverage campaign (UCC); Thies and Dakar had their UCC after the fieldwork for this study.

Results from the sleeping space questionnaires complemented the qualitative findings. Figure  2 presents the data related to net drying practices by region. Of those nets that had ever been washed (n = 252), the majority (70.2%) were dried in the sun. In Saint Louis and Thies, nets were nearly always dried in the sun, which may reduce the effectiveness of the insecticide. This was less common in Dakar and Kolda.

### Net retreatment

A minority of participants mentioned net retreatment as an essential component of net care, often in tandem with washing. Most frequently, respondents stated that they could take the net to the *Service d‘Hygiène* or health facility for re-impregnation or that someone would come to their community to retreat the nets. Those who treated the nets were described as wearing gloves and masks to protect themselves from the product and a few respondents mentioned that they were afraid of the effects of the insecticide.

R*: Yes, they arrive with the new mosquito nets in the small packets. On the interior of the packet, there are often these sachets.*

E*: Ah, these are the sachets that are used?*

R*: Yes, these are the tablets that you put into the basins of water. They wear the gloves and block their nostrils because the product is dangerous after they wash the nets. After washing, they dry them in the sun for two days to diminish the dose of the product until it has diminished enough and you can hang it. But you do not dare to wash at that moment and hang at that moment to pass the night. This is not good for anyone.*

-IDI respondent, Saint Louis rural

Some people reported that they could no longer find supplies for retreatment or currently did not know where they could get their nets retreated.

R*: Yes but the re-impregnation was easy in that if it was accessible, you could go at any moment and re-impregnate, but on the condition that someone told us where to do it. For example, if someone could go to the health posts to find the retreatment, that would be easier but we do not know where to go for retreatment.*

-IDI respondent, Dakar rural

### Seasonal storage of nets as method of care

Some respondents mentioned that in order to take good care of their nets, they used them only in the rainy season when mosquitoes were highly visible. In doing so, they felt that they were preserving their nets for the period they were needed most. Some of the respondents mentioned putting nets away into bags to save them for the rainy season.

*It (the mosquito net) comes at the moment when there are mosquitoes. When there are no more at the time, you fold it well and you save it in a place where the rats cannot spoil it…where the insects…And one day, you check where you put it but you should not wait until the time when the mosquitoes come. You need to take care of it like you take care of your soul because as I told you, health has no price.*

-IDI respondent, Thies rural

### Person responsible for net care

Respondents generally agreed that wives were responsible for washing nets, and that individuals were responsible for handling and storing their own nets. Women in general were tasked with washing and caring for nets. Respondents did distinguish between having adults and children care for nets and indicated that it was most appropriate for adults to do so since children can tear the nets when they are in a hurry.

### Perceptions of those who do not care for nets

Respondents were asked why some people might not care for their nets and typically attributed this to negligence, laziness or ignorance. They felt that these people do not value nets and were therefore putting themselves and their families at risk.

*It is just laziness (that keeps people from caring for their nets). It may also be lack of know-how and ignorance.*

-IDI respondent, Saint Louis urban

### Net repair

According to the IDI and FGD data, respondents most commonly stated that they would repair their nets by sewing with a needle and thread. Most of these respondents said that they would do this themselves but a few said that they would bring it to a tailor for repair. The second most commonly mentioned method of repair was that of tying knots to close the holes. A few respondents said that they would use pieces of another mosquito net or find another piece of fabric to repair the net by patching. Results from the sleeping space questionnaires provided information on 100 nets with evidence of repair. According to the data, 62% were repaired with knots, 36% were repaired by a needle and thread, and 2% were repaired by other means.

### Perceived benefits of repairing a net

Protection from malaria was by far the most commonly mentioned advantage for repairing mosquito nets. Several respondents said that it is better to protect oneself with a net with some small holes than to sleep without one.

R*: The advantage of repair, it’s to protect against the bites of mosquitoes if, as I have said, you don’t have the means to procure a new one. You know that if the mosquitoes bite you frequently, you risk falling ill with malaria. The bite of a mosquito is the mode of malaria transmission. When you do not have a new net and the old one has holes or is torn, you must sew it until you find another.*

-IDI respondent, Kedougou, female

Some respondents said that once a net is repaired, it becomes just as efficient as a new net for protection against mosquitoes.

E*: A sewn net and a new one- is there a difference?*

R*: There is no difference because the mosquitoes can no longer pass through. If you repair, the mosquitoes cannot pass and if it is new also, they can’t pass.*

IDI respondent, Dakar, female

E*: According to you, is it useful to sew nets?*

R*: Yes, it is useful because if you sew, they become new because the mosquito net is not something that can tear quickly because we use it during the rainy season and after, when there are no more mosquitoes, we save the net well.*

-IDI respondent, Ziguinchor, male

### Barriers to net repair

Several participants said that there is no use in a torn net and that it is preferable to acquire a new one rather than repair a torn net.

*If it is very worn, even if sewn, it will no longer be useful.*

-IDI respondent, Ziguinchor, female

Other frequently mentioned challenges regarding net repair were that repair is difficult or impossible and that repairing nets can cause more holes or damage by making them weak. The fragility of nets that have been repaired can make it easy for them to tear again. Therefore, net repair was seen by many respondents as a temporary measure that would postpone the inevitable need to purchase a new net.

E*: Now if you have to repair it, how will you repair it?*

R4*: The repair will aggravate the holes because in the mosquito net, there are already holes. If you use the needle to sew, when pulling, you will open another gap. Therefore, me, I think that if it is torn the only solution is the trash.*

-FGD respondent, Thies, female

A substantial number of respondents stated they would prefer to throw the net away instead of repairing it. They gave different reasons for this decision, including not having enough time to repair and not wanting to waste the money to repair when that money could be invested in a new net. One woman from Ziguinchor stated that:

*There are no advantages to repairing a net. If I do it, it’s only due to poverty.*

-IDI respondent, Ziguinchor, female

## Discussion

Malaria control efforts to date have largely been concerned with the distribution and promotion of net use, with less emphasis on the prevention and reparation of damage to the net. Existing evidence on durability, however, suggests that in different contexts nets have variable useful lifespans [6, 8, 10–12]. In some places, the useful life of a net may be shorter than three years [6, 8, 11, 12], perhaps due to environmental context and net care and repair practices [8, 17, 18]. Because many of the perceived causes of damage in this study are preventable and most damage is reparable, it is useful from a public health and cost-effectiveness perspective to build interventions and SBCC programmes to promote the extension of net life. The results of this research provide new insights into community perceptions and actions regarding net care and repair and are useful for guiding the development of net care and repair interventions.

**Figure 3:**
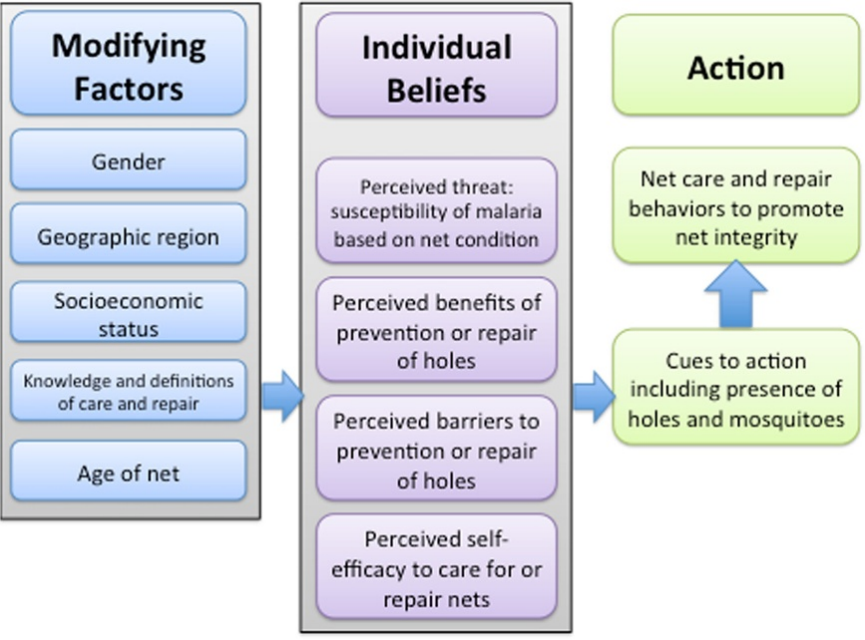
**Health Belief Model for net care and repair.**

The conceptual model below (Figure 3) is an adaptation of the HBM applied to the behaviours of net care and repair. The HBM posits that for people to change their behaviour, they must feel threatened by their current situation (perceived severity and susceptibility), believe that a change in behaviour will have an overall favourable outcome despite the costs (perceived benefits), and believe that they are competent (self-efficacy) to overcome any perceived barriers to change and take action [29]. According to HBM, behaviour change is especially likely in the context of cues to action, or situations that trigger behaviour [29]. Theoretical models are useful in guiding evidence-based SBCC and intervention approaches. The results previously presented can be understood through the HBM framework and used to develop a social and behaviour change communication strategy.

In this model, potential modifying factors are included as they are likely to affect the individual-level beliefs of perceived threat of malaria, perceived benefits of net care and repair, perceived barriers of net care and repair, and self-efficacy to care for and repair nets. These four components affect the likelihood that people will take action to promote the integrity and durability of their nets.

### Perceived threat

In this study, respondents reported that their primary motivation for net care and net repair was protection from malaria. While the study did not specifically investigate the perceived threat of malaria to respondents, other studies have demonstrated that the perceived threat of malaria is generally high in malaria endemic countries [30]. This suggests that perceived susceptibility and/or the perceived severity of the disease is high. In this context, a communication strategy highlighting the danger of malaria would be unlikely to be effective in Senegal. Respondents also perceived an increased threat of malaria when using a net that is in poor condition. In another paper from this study [20] respondents generally preferred to get a new net when their existing net had too many holes or when mosquitoes had entered the net because they believed they were at a higher risk of malaria. Thus, while the ideal behaviour of repair is not always being practiced, the perceived threat of malaria due to the poor condition of a net appears to remain.

### Perceived benefits of care and repair

While the prevention of malaria was the most commonly mentioned benefit of having a net in good condition, several respondents mentioned that there was a social value placed on a well cared for net. Respondents attributed lack of care to laziness, negligence and ignorance. The Senegal findings are similar to those of other studies [13, 24] showing social pressures against having a net that is unclean. This negative view of nets in poor physical condition could be leveraged in SBCC activities to promote the positive qualities of those who care for their nets. Care and repair of nets could be linked to other regular household cleaning and maintenance routines to build and reinforce habits of caring for nets, as part of a neat, clean household. Other reported benefits of net care and prevention of holes included cost savings from averting illness, sleeping better at night, and the need to extend net life due to uncertainty of when one could expect a new net. Getting a good night’s sleep has been demonstrated as an important motivator of net use [27] and could be further promoted to encourage net care and repair behaviours. To build positive social pressure for net repair, SBCC programmes could highlight these benefits of net care and repair and publicly portray these champions of net integrity.

### Perceived barriers to net care and repair

There were a number of perceived barriers to net care and repair, all of which could be addressed through SBCC programming and interventions. Small children and pests were reported to contribute to holes and tears. Knowledge about how to care for nets varied considerably. Some respondents suggested that to care for nets, they should not be used in seasons with few mosquitoes. Furthermore, net washing and drying practices were variable and often included the use of harsh detergents such as Omo and drying nets in the sun, two practices that may be harmful to nets. Improvement of these practices should be a targeted goal of future SBCC campaigns.

Barriers to net repair appeared to be more significant. When nets were torn, people generally preferred to seek out a new net rather than repair their existing net [20]. This was largely related to the challenges of repair, and the feeling that repair could cause even more holes or result in a net that was weaker than before. Furthermore, respondents reported a lack of time and money for materials to repair their nets. Communication initiatives should address these attitudes by describing small doable actions that households can take, such as repairing small holes early on, and modelling how quickly and easily holes can be repaired with materials already available in the household. Since this study found that people’s view that net repair at best postponed and at worst contributed to the degradation of nets, SBCC programmes may need to ‘rebrand’ net repair as a positive behaviour, rather than one that causes further damage.

### Self-efficacy for net care and repair

Self-efficacy is defined as ‘the conviction that one can successfully execute the behaviour required to produce the outcomes’ [31]. This study did not ask directly about self-efficacy in either the qualitative or quantitative instruments. While one might assume that the high reported levels of net care would imply high efficacy for these behaviours, it is not possible to report this for certain. Respondents generally reported high levels of net care by storing them well to avoid holes, washing, drying, and using them seasonally. Self-efficacy for net repair, however, may have been lower, with fewer reported repairs made and many reported financial and time-related barriers to repair. The results of this study suggest some important dimensions of self-efficacy for net care and repair that should be measured in future work on behaviour change towards care and repair. It would be useful to understand the self-efficacy of respondents to conduct the ideal care and repair behaviours as well as those that they perceive to be important but that may not match public health guidelines. For example, while public health practitioners suggest the use of nets throughout the year, several respondents discussed caring for nets by storing them during the dry season for use when the mosquitoes are more prevalent. Future self-efficacy measures would seek to understand the degree of confidence in this behaviour and how this confidence might be redirected to other care and repair behaviours. Furthermore, since children were cited as major sources of net damage, it would be helpful to understand participants’ self-efficacy in preventing damage either through educating children (admittedly difficult with younger children) or through different storage practices. Likewise, in settings where pest-related damage is high, it would be important to measure self-efficacy to store nets away from pests and to control pest populations.

### Cues to action

Based on these results, cues to action for net care and repair include the visual condition of the net as well as the likelihood that a net will be replaced. The finding that in some cases, a lack of knowledge about when nets would be distributed again resulted in improved care practices has implications for public health programming. It is important that programmes provide the message that mass distribution campaigns may not always be possible and that people should protect themselves through the care and repair of the nets that they have. Washing practices appeared to be associated with age of the nets, or time since the universal coverage campaign (UCC). Cues to action for washing were fairly clear, with respondents reporting that they should wash nets when they became obviously dirty. Respondents in Louga and Ziguinchor largely reported that they had not yet washed their nets, which they had just received during the 2012 UCC. Nets were washed more frequently in the regions that received the UCC in 2010, and where SBCC messages on washing and repair were not included in activities and job aids. However, cues to action in other areas of net care such as net storage and net handling were less clear. While the presence of holes was a motivation for repair, it was not clear how many holes or the size of holes that would warrant net repair. A targeted SBCC approach would need to highlight and create cues to action, such as the presence of small holes needing to be repaired quickly, tying up the net during the day as a regular part of the morning routine. Communication will also need to focus on promotion of benefits and minimizing barriers to net repair, making these behaviours into small, do-able actions.

### Study limitations

This study had several important limitations. First, the study utilized primarily qualitative methods and captured the experiences and perceptions of a small number of participants. Participants were purposively selected to reflect the experiences of people throughout many regions in Senegal. While the study provides in-depth insights into the knowledge and attitudes of these respondents, it is limited in that these experiences cannot be generalized to other populations or contexts.

In addition, since the sleeping space questionnaires were conducted in several sleeping spaces within households involved in the study, the data from these questionnaires were highly correlated. The data were used to supplement the self-reported data and cannot be used to draw conclusions about net care and repair practices across Senegal due to a the sampling design and lack of statistical power.

Specifically in Louga, respondents assumed that the data collectors had been involved in the net distribution. Although the team stated that the researchers were not associated with the distribution, it is possible that the respondents may have continued to believe that the data collectors were affiliated with the net distribution. This may have resulted in a courtesy bias, where respondents provided answers that they thought would be favourable to the team.

Finally, this study lacked measures of self-efficacy for net care and repair behaviours and is not able to provide estimates of the respondents’ confidence in net care and repair. While the high levels of net care suggest that lack of efficacy did not appear to be a barrier, it remains unclear whether respondents feel completely comfortable and able to conduct net care and repair behaviours.

Despite these limitations, this research provided important insights into care and repair barriers, motivations, and perspectives in several regions in Senegal, a country with a history of net distribution and a relatively strong culture of net use. Additional research on net care and repair from other country contexts is necessary to provide insights into whether these beliefs and behaviours occur in other environments. In addition, qualitative data should be triangulated with observational and quantitative data in order to quantify and better understand these practices. Pretesting messages and approaches will be useful in order to define appropriate, evidence-based approaches to promote care and repair. Finally, it will ultimately be important to understand whether SBCC campaigns can affect behaviours and whether this affects the outcomes of improved net integrity and extended/prolonged net lifespan.

## Conclusion

The results of this research provide us with important information on perceived causes of net damage as well as the motivations and barriers to net care and repair in Senegal. Nets are typically damaged by behaviours related to daily use. In addition, respondents reported varying frequencies and practices of net washing, drying and storage. Net repair was seen as challenging and as only delaying the inevitable degradation of the net. The HBM provides a useful lens for these results: in Senegal, perceived threat of malaria is high and households weigh the benefits of net care and repair against the perceived benefits. The HBM suggests that in this context, if benefits are greater than barriers and if the person has the self-efficacy to carry out the desired behaviour, behaviour change is more likely. A cue to action may be needed to spur the person to action. This research therefore suggests that in this context, SBCC programmes should promote benefits of net care and repair and provide people with small doable actions for overcoming barriers. Furthermore, SBCC interventions can provide cues to action, re-inforcing social norms around the benefit of well-maintained nets and providing reminders to repair holes as soon as possible. Additional research would be beneficial for understanding the role of self-efficacy relative to net care and repair and to further characterize these findings and their applicability to other settings.
